# SERTAD1 Sensitizes Breast Cancer Cells to Doxorubicin and Promotes Lysosomal Protein Biosynthesis

**DOI:** 10.3390/biomedicines10051148

**Published:** 2022-05-17

**Authors:** Hai Anh Nguyen, Son Hai Vu, Samil Jung, Beom Suk Lee, Thi Ngoc Quynh Nguyen, Hyojeong Lee, Hye-gyeong Lee, Davaajargal Myagmarjav, Taeyeon Jo, Yeongseon Choi, Myeong-Sok Lee

**Affiliations:** Department of Biological Science, Sookmyung Women’s University, Seoul 04310, Korea; anhnh1@hus.edu.vn (H.A.N.); vh.son@hutech.edu.vn (S.H.V.); samiljung@sookmyung.ac.kr (S.J.); min9996@sookmyung.ac.kr (B.S.L.); ntnquynh07@sookmyung.ac.kr (T.N.Q.N.); 1642971@sookmyung.ac.kr (H.L.); lhg0914@sookmyung.ac.kr (H.-g.L.); davaa@sookmyung.ac.kr (D.M.); 1931676@sookmyung.ac.kr (T.J.); 2131587@sookmyung.ac.kr (Y.C.)

**Keywords:** breast cancer, anti-cancer drugs, anoikis, autophagy, SERTAD1, lysosomal biogenesis

## Abstract

Acquired chemoresistance of tumor cells is an unwanted consequence of cancer treatment. Overcoming chemoresistance is particularly important for efficiently improving cancer therapies. Here, using multiple lines of evidence, we report the suppressive role of SERTAD1 in apoptosis/anoikis. Among various breast cancer cell lines, higher SERTAD1 expression was found in MCF7 and MDA-MB-231 in suspension than in adherent cell culture. We revealed an unexpected phenomenon that different types of cell deaths were induced in response to different doses of doxorubicin (Dox) in breast cancer cells, presumably via lysosomal membrane permeabilization. A low dose of Dox highly activated autophagy, while a high dose of the chemotherapy induced apoptosis. Inhibition of SERTAD1 promoted the sensitivity of breast cancer cells to Dox and paclitaxel, leading to a significant reduction in tumor volumes of xenograft mice. Simultaneously targeting cancer cells with Dox and autophagy inhibition successfully induced higher apoptosis/anoikis. The novel role of SERTAD1 in maintaining cellular homeostasis has also been suggested in which lysosomal contents, including LAMP1, LAMP2, CTSB, and CTSD, were reduced in SERTAD1-deficient cells.

## 1. Introduction

Cancer is the disease of cellular abnormalities, which can be classified by the type of originated tissues. Breast cancer is the most common cancer, and the second leading cause of death in diagnosed women [1]. Beside the uncontrolled fashion of proliferation, what truly makes cancer hard to cure is its ability to spread throughout the body by entering the circulatory system. Generally, upon detachment from the extracellular membrane, cells undergo a specific type of programmed cell death (PCD) called anoikis which plays a crucial role in maintaining cellular homeostasis and avoiding the distribution of cells to the wrong location [2]. One of the thorny issues of concern to cancer researchers is the resistance of cancer cells against anti-cancer drugs; thus, understanding the activation of prosurvival-signaling cascades and cell death inhibition upon chemotherapeutic treatments appears to be crucial to developing effective treatments [3].

Autophagy promotes the survival of cancer cells by shielding them from apoptosis, prompting the need to use autophagy inhibitors as clinical cancer treatments [4]. Unlike apoptosis, autophagy acts as a double-edged sword in the cancer setting by exhibiting both tumor promotion and suppression properties [5]. Simultaneously targeting PCDs and autophagy can be an effective strategy to eliminating anti-cancer drug resistance. Materials destined for degradation enter lysosomes via several intracellular transport processes, such as autophagy, endocytosis, and phagocytosis. Other than being an indispensable part of autophagy, lysosomes have essential functions in numerous cellular processes, such as cell death, inflammasome activation, and immune response [6]. Lysosomal membrane permeabilization (LMP) is a major kind of lysosomal-dependent cell death. The process is characterized by aberrant changes in pH of the cytosol and lysosomes and in iron homeostasis [6]. Eventually, LMP causes the release of the lysosomal contents to its surrounding cellular environment upon receiving noxious stimuli. A massive breakdown of the lysosomes would lead to uncontrolled necrosis. Meanwhile, partial and selective leakage of lysosomal contents may trigger other cell death pathways [7].

Lysosome biogenesis is a vital intracellular process to control the number and size of lysosomes needed for cellular degradation [8]. The integration of lysosomal protein biosynthesis and endosome–lysosome trafficking is indispensable for lysosome biogenesis. Lysosomal hydrolases are produced and modified in the endoplasmic reticulum and the Golgi complex, which undergo further modification in their biological structure and trafficking to be delivered to the designated place [8,9]. Advanced understanding of lysosome biology is critical regarding the roles of lysosomes in cellular homeostasis, development, and aging [8]. There is a number of transcription factors, repressors, and epigenetic regulators closely coordinated to regulate lysosomal biogenesis, but the role of other proteins involved in the process is not fully characterized.

SERTAD1 (TRIP-BR1/p34^SEI-1^/SEI1) has emerged as an important protein in diverse biological processes [10]. SERTAD1 has been widely accepted as an oncogene, and its upregulated expression is found in multiple malignancies [11,12]. SERTAD1 also affects cancer cell survival and tumorigenesis by inducing ubiquitination and degradation of PTEN in a NEDD4-1-dependent manner [13]. Findings from our group have strengthened the oncogenic trait of SERTAD1 as it affects multiple PCDs under stressful conditions and metastasis [10,11,14,15]. The role of SERTAD1, however, in anti-cancer drug resistance of breast cancer cells and regulation of cellular homeostasis has not been understood.

In this study, we examined how SERTAD1 sensitized cancer cells to anti-cancer drugs and the role of SERTAD1 in lysosomal protein biosynthesis was also investigated.

## 2. Materials & Methods

### 2.1. Cell Culture and Treatment Preparation

Cell lines used were purchased from the American Type Culture Collection (ATCC). Breast cancer cell lines, including MCF7, MDA-MB-231, MDA-MB-453, T47D, MDA-MB-468, BT20, BT549, SKBr3, and HS578T, were cultured in Dulbecco’s Modified Eagle’s Medium (DMEM, WelGENE Inc., Gyeongsan-si, South Korea) supplemented with 10% fetal bovine serum (FBS) (Gibco BRL, Waltham, MA, USA) and 1% antibiotic-antimycotic solution (Gibco BRL, Cat#15240-062, USA). Normal human MCF10A mammary epithelial cells were grown in DMEM/F12 medium (Invitrogen, Cat#11330-032, Waltham, MA, USA), supplemented with 20 ng/mL of epidermal growth factor (EGF) (Sigma-Aldrich, Cat#E9644, St. Louis, MO, USA), 100 ng/mL of cholera toxin (Sigma-Aldrich, Cat#C-8052, USA), 10 μg/mL of insulin (Sigma-Aldrich, Cat#I-9278, USA), 0.5 mg/mL of hydrocortisone (Sigma-Aldrich, Cat#H-0888, USA), 5% horse serum (Invitrogen, Cat#16050-122, USA), and 1% antibiotic-antimycotic solution (Biowest, Cat#L00100, Nuaillé, France). MCF7 stable cell lines with wild-type SERTAD1 (MCF7^WT-SERTAD1^) or knock-down SERTAD1 (MCF7^KD-SERTAD1^) were established in our previous study [11]. Cells were kept in a humidified atmosphere containing 5% CO_2_ at 37 °C. PolyHEMA-coated plates were prepared by dissolving 10 g/mL polyHEMA (Santa Cruz Biotechnology, Inc., Cat#sc-253284, Dallas, TX, USA) in 95% ethanol and then adding 3–4 mL of the solution to 100 mm Petri plates or 1.3 mL of the solution to 60 mm Petri plates (SPL Life Sciences, Gyeonggi-do, South Korea). Doxorubicin (Sigma-Aldrich, Cat#D2975000, USA) and chloroquine (Sigma-Aldrich, Cat#C6628, USA) were prepared by following the manufacturer’s recommendation.

### 2.2. Cell Viability Assays

Cell viability was assessed by trypan blue staining, MTS, and CCK assay. Briefly, cells were plated onto polyHEMA-coated or non-coated 96-well plates for 24 h and then the cells were treated with or without treatments for 24 h. MTS assay was carried out using Cell Titer 96 non-radioactive cell proliferation assay kit (Promega, Cat#G4000, Madison, WI, USA). The CCK assay was performed using D-Plus^TM^ CCK kit (Dongin, Cat#CCK-1000, Seoul, South Korea). The Ledetect 96-well plate reader (Labexim, Lengau, Austria) was used for fluorescent determination.

### 2.3. Western Blot Analysis

Cells were collected by centrifugation, then washed with ice-cold phosphate-buffered saline (PBS), and lysed in radioimmunoprecipitation assay (RIPA) lysis buffer (50 mM Tris, pH 7.5; 48 mM NaCl; 1% Triton X-100; and 1 mM EGTA) supplemented with 0.5 mM Na_3_VO_4_, 1 mM DTT, and 2 µL pre-made protease inhibitor cocktail III (AG Scientific, Cat#P-1512, San Diego, CA, USA) per mL of RIPA buffer. The protein concentration was quantified using Bradford assay. Blotting samples were prepared by adding Sample Buffer (Laemmli’s 5x, ecocell, Gyeonggi-do, South Korea), were denatured by boiling for 10 min, and were then subjected to SDS-PAGE and transferred onto an Immobilon-P^®^ polyvinylidene fluoride transfer membrane (EMD Millipore, Cat#IPVH00010, Burlington, MA, USA). Membranes were incubated with each corresponding antibody, and immunodetection was carried out using the Claro™ Mucho ECL solution (BioD, Cat#HQS070, Gyeonggi-do, South Korea). The antibodies used in this study were purchased as follows: anti-SERTAD1 (Enzo Life Sciences, Cat#ALX-804-645, Farmingdale, NY, USA), anti-LC3B (Enzo Life Sciences, Cat#ALX-803-082, USA), anti-PARP (Cell Signaling Technology, Cat#9542, Danvers, MA, USA), anti-cathepsin A (Bioworld, #BS60829, Visalia, CA, USA), anti-cathepsin B (Bioworld, Cat#BS3536, USA), anti-cathepsin D (Bioworld, Cat#BS90201, USA), anti-LAMP1 (Santa Cruz Biotechnology, Cat#sc-20011, USA), anti-LAMP2 (ThermoFisher, Cat#PA1-655, Waltham, MA, USA), and β-actin (Santa Cruz Biotechnology, Cat#sc-47778, USA). β-actin was used as a loading control. The horseradish-peroxidase-conjugated secondary antibodies were anti-rabbit (Cell Signaling Technology, Cat#7074S, USA), anti-mouse (Santa Cruz Biotechnology, Cat#sc-516102, USA), and anti-goat (Santa Cruz Biotechnology, Cat#sc-2020, USA). The results of the Western blot analysis were semi-quantified using ImageJ software (version 1.51u; https://imagej.nih.gov/ij/, accessed on 5 June 2019) supplied by the National Institutes of Health (Bethesda, MD, USA).

### 2.4. Immunoflorescene (IF) Staining for Cells

Cells were grown on confocal dishes (Coverglass-Bottom Dish, SPL. Cat#100350, Gyeonggi-do, South Korea) at 5 × 10^4^ cells/mL for 24 h. Subsequently, cells were treated with 1 µM or 5 µM of doxorubicin (Dox) for 24 h. Then the cells were fixed in 4% formaldehyde for 15 min, washed thrice with PBS, permeabilized with 0.3% Triton X-100 for 1 h, and then incubated with the indicated antibodies overnight at 4 °C. Alexa Fluor^®^ 555 (Abcam, Cat#ab150074, Cambridge, UK) were diluted 1:100 in IF blocking buffer. Nuclei were stained with DAPI (Invitrogen, Cat#P36931, USA) for 10 min. Samples were imaged using a laser-scanning confocal microscope (Nikon A1, Melville, NY, USA) and fluorescence was analyzed. Three to four images from different areas were taken per plate. ImageJ was used to quantify the results.

### 2.5. Mitotracker Staining

Cells were plated at 5 × 10^4^ cells/mL on confocal dishes and were allowed to grow overnight. The following day, the cells were treated with either 1 µM or 5 µM of Dox or vehicle for 24 h. Then 200 nM MitoTracker Deep Red FM (Invitrogen, Cat#M22426, USA) was added to the cells for 30 min. Cells were fixed in 4% formaldehyde for 30 min and washed twice with PBS. Samples were imaged using a laser-scanning confocal microscope (Nikon A1, USA) and fluorescence was analyzed. MitoTracker was excited at 644 nm and emission spectra were collected from 665 nm. Three to four images from different areas were randomly captured per plate for analysis. ImageJ was used to quantify the results.

### 2.6. Lysotracker Staining

Cells were plated at 5 × 10^4^ cells/mL onto confocal dishes and incubated overnight. Cells were treated with either dDox or vehicle for 24 h, after that 100 nM LysoTracker^®^ Deep Red (Invitrogen, Cat#L12492, USA) in phenol-free medium was added to cells and incubated for 90 min. The cells were washed twice with PBS and were imaged using a laser-scanning confocal microscope (Nikon A1, USA). The LysoTracker was excited at 647 nm and the emission spectra were collected from 668 nm. Three to four images were randomly taken per plate for analysis. ImageJ was used to quantify the results.

### 2.7. Acridine Orange (AO) Staining of Lysosomes

AO, a lysosomotropic metachromatic fluorochrome dye, labels acidic vesicular organelles (AVOs), such as autolysosomes, and is also used in autophagy assays. The intensity of red fluorescence is proportional to the degree of acidity and the acidic compartment volume. Briefly, MCF7 (5 × 10^4^ cells/mL) were plated in sterilized round-type cover glasses (Deckglaser, Cat#0111520, Freiburg, Germany) or poly-HEMA-coated plates. The next day, the cells were incubated for 24 h with the indicated concentrations of Dox. After the treatment course, the cells were stained with 2.5 μg/mL AO (Invitrogen, Cat#A1301, USA) for 15 min at 37 °C. AVOs formed in cells were immediately observed using a laser-scanning confocal microscope (Nikon A1, USA) and fluorescence was analyzed at 20X magnification. Red fluorescence quantification was performed using Image J.

### 2.8. Real-Time Polymerase Chain Reaction (RT-PCR)

For mtDNA quantification, total DNA was extracted using HiYield PlusTM Genomic DNA Mini Kit (Real Biotech Corporation, Cat#QBT100, Taiwan). The resulting DNAs were quantified by real-time PCR using 2X SYBR Green PCR master mix (ThermoFisher, Cat#4367659, USA), 0.2 pM primers, sterile H_2_O, and 2 µL DNA extract on the StepOnePlus™ (Applied Biosystems, Waltham, MA, USA), using the following primers: mtDNA-Fw: 5′-AGGACAAGAGAAATAAGGCC-3′ and mtDNA-Rev: 5′-TAAGAAGAGGAATTGAACCTCTGACTGTAA-3′, and GAPDH-Fw: 5′-CTGGGCTACACTGAGCACCAG-3′ and GAPDH-Rev: 5′-CCAGCGTCAAAGGTGGAG-3′.

### 2.9. Breast Cancer Gene-Expression Miner v4.5 (bc-GenExMiner v4.5)

The Breast Cancer Gene-Expression Miner v4.5 (http://bcgenex.ico.unicancer.fr, accessed on 14 March 2020) [14], a DNA microarray (n = 10,716) database, can be used to analyze correlation, expression, and prognosis. The association between DNA expression of SERTAD1 versus REACTOME_MITOCHONDRIAL_BIOGENESIS gene set, LYSOSOME gene set (GO:0005764), and LYSOSOME_ORGANIZATION_AND_BIOGENESIS gene set (GO:0007040) (https://www.gsea-msigdb.org, accessed on 14 March 2020) of PR+/ER+, PR+/ER−, PR−/ER+, and PR−/ER− patients was evaluated [15,16].

### 2.10. Mice Genotyping and Isolation of Mouse Embryo Fibroblasts (MEFs)

Knocking out SERTAD1 mice (RRID: MGI:4437096) in a C57BL/6 genetic background was a kind gift from Dr. Pingbo Huang (Hong Kong University of Science and Technology, Hong Kong, China). Genotyping mice was done as previously described [17] using the following primers: SERTAD1 wild-type (WT) forward primer: 5′ CCATCCCCAGCATCAAATACACCA 3′, SERTAD1 wild-type reverse primer: 5′ CTCCCGCTTGCGCTTCAGACCTT 3′, and SERTAD1 knock-out (KO) reverse primer: 5′ CATAGCCTGAAGAACGAGAT 3′. SERTAD1 wild-type and knockout C57BL/6 mice were pregnant about 13.5 days, and then the embryos were dissected and decapitated. Internal organs were removed. The tissues were washed with cold PBS and then cut into small pieces and incubated with Trypsin/EDTA for 15 min at 37 °C. Non-adherent cells were removed, and adherent cells were maintained in DMEM media supplemented with 10% FBS, 1% 200 mM L-glutamine after each incubation.

### 2.11. Xenograft Mice Establishment

Five-week-old female BALB/c nude mice were acclimatized for three days upon arrival. Twenty-four mice were randomly divided into four groups: vehicle treatment (i) MCF7^WT-SERTAD1^ (ii) MCF7^KD-SERTAD1^; Dox treatment, (iii) MCF7^WT-SERTAD1^, and (iv) MCF7^KD-SERTAD1^. The cells (1 × 10^7^ cells/mice) were prepared in sterile PBS and mixed (1:1) with Matrigel^®^ Basement Membrane Matrix, Phenol Red-free, LDEV-free (Corning, Cat#356237, Corning, NY, USA) and then subcutaneously injected to the left thigh. When tumor size reached approximately 100 mm^3^, the mice were intravenous injected with 5 mg/kg Dox once every three days over a period of two weeks. Tumor size was measured every three days, and the tumor volumes were calculated using the formulation: 0.523 × length × width^2^.

### 2.12. Survival Rate Study

Xenograft mice were established as described above. Tumor size was measured every three days after implantation, and the tumor volumes were noted and calculated. Mice were counted as dead once they died in a natural way, or the tumor burden exceeded 20 mm in any direction. These parameters were used to construct a Kaplan–Meier survival curve.

### 2.13. IF Staining for Tumors

The xenograft mice were sacrificed, and the tumors were freshly collected to perform the experiment. Then, formalin-fixed and paraffin-embedded sections of 5 µm thickness were dried at 60 °C for 30 min, deparaffinized in xylene, rehydrated through graded alcohols, and immersed for 15 min in PBS buffer. For antigen retrieval, the sections were microwaved in 0.01 M citrate buffer (pH 6.0) for 20 min. After that, the endogenous peroxidase activity was blocked with 0.3% hydrogen peroxidase in methanol for 30 min. The sections were incubated for 60 min with 5% BSA and 0.3% Triton X-100 in PBS-T to block nonspecific staining and then incubated with indicated primary antibodies overnight. Alexa Fluor^®^ 555 (Abcam, Cat#ab150074, UK), Alexa Fluor^®^ 647 (Abcam, Cat#ab150115, UK), and Alexa Fluor^®^ 647 (Abcam, Cat#ab150131, UK) were diluted in IF-blocking buffer. The specimens were washed with PBS, and then the nuclei were stained with DAPI (Invitrogen, Cat#P36931, USA) for 20 min. The slides were mounted with VECTASHIELD Antifade Mounting Medium for Fluorescence (Vector Laboratories, Cat#H-1000, Burlingame, CA, USA). The samples were imaged using a laser-scanning confocal microscope (Nikon A1), and fluorescence was analyzed. Three to four images were randomly taken per slide for analysis.

### 2.14. Statistical Analysis

Data are presented as the means ± standard deviation from three independent experiments. Statistical analysis was performed using Student’s *t*-test to compare two different groups or one-way analysis of variance, followed by Bonferroni’s multiple comparisons test, to compare multiple groups. SPSS statistics version 23 was used to analyze data (IBM Corporation, Armonk, NY, USA). *p* < 0.05 was considered as statistically significant.

## 3. Results

### 3.1. SERTAD1 Suppresses Anoikis Resistance in Breast Cancer Cells

**Figure 1:**
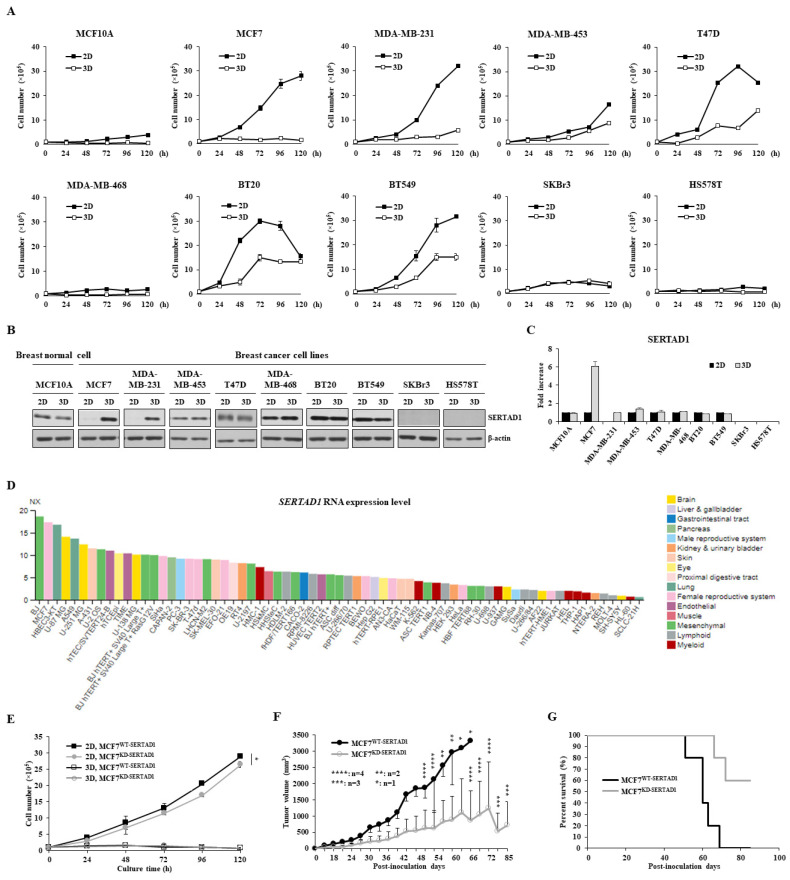
Cell viability of various breast cell lines in response to monolayer culture and suspension culture. (**A**) Cell proliferation of breast cell lines in 2D and 3D culture systems. Cell viability of each cell line was measured using trypan blue-staining assay every 24 h. Data are presented as mean ± SD based on three independent experiments. (**B**,**C**) Western blot analysis of SERTAD1 expression in 2D and 3D cell culture systems. Cells were cultured for 48 h and then lysed for immunoblotting. Data are representative of three independent experiments with similar patterns. (**D**) *SERTAD1* mRNA expression overview in human cancer cell lines in the Human Protein Atlas [18]. Data summary images were obtained from: https://www.proteinatlas.org/ (version 20.1), via: https://www.proteinatlas.org/ENSG00000197019-SERTAD1/cell (accessed on 25 July 2020). (**E**) Cell growth of the two stable cell lines—MCF7^WT-SERTADl^ and MCF^KD-SERTADl^ in 2D and 3D culture conditions using trypan blue-staining assay. Data are presented as the mean ± SD from three independent experiments. * *p* < 0.05. (**F**,**G**) Tumor volumes and survival rates of xenograft mice implanted with MCF7^WT-SERTADl^ or MCF^KD-SERTADl^.

Normal breast epithelial and nine breast cancer cell lines were cultured to assess the difference in cell growth in both adhesion (2D) and suspension (3D) culture conditions in 120 h. A huge difference in cell viability was observed between adherent and suspension culture in several breast cancer cell lines, especially MCF7, MDA-MB-231, T47D, BT20, and BT549. Among them, MCF7 and MDA-MB-231 displayed the most contrast cell growth as their cell proliferation remained virtually unchanged in the 3D culture (Figure 1A). As SERTAD1 is found to be upregulated in cancer cells upon stressful stimuli [11], we speculated that SERTAD1 expression in breast cancer cells may differ under 2D vs. 3D conditions. Indeed, after 48 h, SERTAD1 was notably increased in 3D as compared to 2D cultures in MCF7 and MDA-MB-231 cells, while other cell lines did not show the same pattern (Figure 1B,C). The transcriptional profile of *SERTAD1* in other cancer cell lines from the HPA database also supported that the mRNA expression of *SERTAD1* was significantly upregulated in breast cancer cell lines, especially MCF7 (Figure 1D). MCF7 was then chosen for the next experiments.

We then assessed the proliferative impact of SERTAD1 on MCF7. As shown in Figure 1E, cell proliferation of MCF7^WT-SERTAD1^ is higher than that of MCF7^KD-SERTAD1^ in the 2D culture, but not under the 3D condition. To evaluate the effect of SERTAD1 in vivo, the xenograft model with nude mice was established. We observed that the mean volume of MCF7^KD-SERTADl^ injected tumors was significantly lower than that of MCF7^WT-SERTADl^ injected tumors (Figure 1F). Moreover, a Kaplan–Meier survival curve construction described an increase in the life span of the MCF7^KD-SERTADl^ group compared to the MCF7^WT-SERTADl^ group (Figure 1G). Mice in the control group lasted for 51-69 days after tumor inoculation while MCF7^KD-SERTADl^ implanted mice survived until the end of the experiment. These findings suggest the negative effect of SERTAD1 on the survival rate in the xenograft mice model.

### 3.2. SERTAD1 Suppression Promotes Sensitivity of Cancer Cells to Anti-Cancer Drugs

**Figure 2:**
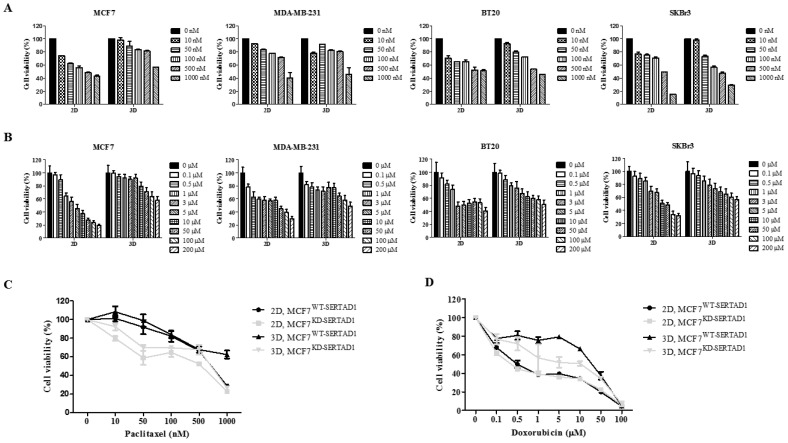
Drug resistance of breast cancer cells against anti-cancer drugs. (**A**,**B**) Various indicated concentrations of ptx and Dox were treated to breast cancer cell lines for 24 h in 2D and 3D conditions. (**C**,**D**) Cell growth of the two stable cell lines—MCF7^WT-SERTADl^ and MCF^KD-SERTADl^ upon treating with various indicated concentrations of ptx and Dox in 2D and 3D culture conditions. Cell viability of each cell line was measured 24 h after cells were treated with ptx or Dox using trypan blue-staining assay. Data are presented as mean ± SD based on three independent experiments.

The role of SERTAD1 in anti-cancer drug resistance is not known. We tested the effect of two anti-cancer drugs, including paclitaxel (ptx) and Dox, against multiple breast cancer cell lines (Figure 2A,B). It was found that MCF7 exhibited the highest resistance to various concentrations of ptx and Dox incubated for 24 h in suspension culture (Figure 2A,B). Two stable cell lines, including MCF7^WT-SERTAD1^ and MCF7^KD-SERTADl^, were exposed to different dosages of the two drugs for 24 h. Both cell lines grown in the 3D culture were more resistant to high concentrations of ptx and Dox compared to the 2D culture (Figure 2C,D). MCF7^KD-SERTADl^ cells in the 3D culture were more sensitive to anti-cancer drugs, even relatively low dose (Figure 2C,D). It has been proposed that ptx kills cancer cells by activating mitotic checkpoint to prevent chromosome mis-segregation and not by inducing apoptosis as Dox [19,20]. In addition, since SERTAD1 has been reported to play roles in three PCDs [11], we selected dDox for further investigation.

**Figure 3:**
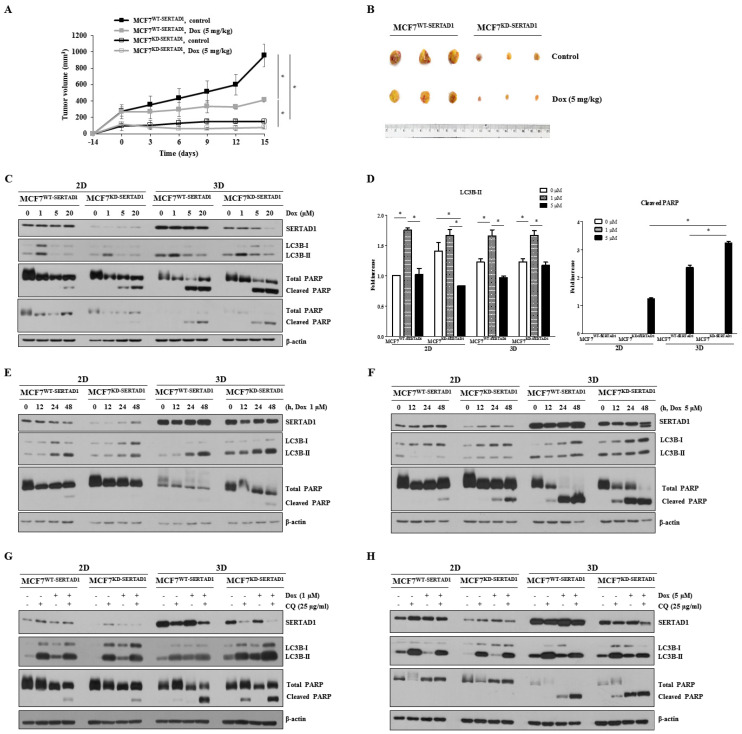
SERTAD1 to promote sensitivity of MCF7 to Dox. (**A**,**B**) Changes in tumor size upon Dox treatment. Data are presented as mean ± SD based on six tumor-bearing animals. Representative tumors (n = 3). (**C**–**F**) Western blot analysis of autophagic and apoptotic markers (LC3B and PARP, respectively) in the two stable cell lines in the presence or absence of treatments in 2D and 3D culture. (**G**,**H**) Single treatment or co-treatment of the autophagy inhibitor and Dox in the presence or absence of SERTAD1. Cells are cultured for 24 h before being subjected to treatments, including either Dox 1 µM or 5 µM or CQ (25 µg/mL). Protein expression of SERTAD1, LC3B-I, LC3B-II, total and cleaved PARP are examined and normalized to β-actin. Data are representative of at least three independent experiments with similar patterns. Blotting results were quantified using ImageJ. * *p* < 0.05.

The susceptibility of SERTAD1-deficient cells to the Dox treatment was also examined in the xenograft mouse model. In accordance with the in vitro results, the MCF7^KD-SERTADl^ injected tumor appeared to proliferate at a slower rate than the wild-type control, and tumor growth was halted in Dox-treated groups compared to mocked treatment over a 15-day course (Figure 3A,B). Taken together, these data suggest that SERTAD1 blockage sensitized breast cancer cells to the anti-cancer drug in both in vitro and in vivo. Apoptosis/anoikis and autophagy are frequently induced to kill or promote survival of cancer cells, respectively, during anti-cancer treatments [21,22]. To elucidate the potential role of SERTAD1 in Dox-induced anoikis resistance, the induction of apoptotic and autophagic markers was checked in dose- and time-dependent mannerd under 2D and 3D culture conditions. We found an increase in activation of LC3B-II only when the cells were treated with 1 µM of Dox while higher tested concentrations strongly induced cleaved PARP as compared with the mocked treatment (Figure 3C,D). Our immunofluorescence results also showed that the number of LC3 puncta was significantly elevated in 1 µM Dox-treated cells compared to the control (Supplementary Figure S1A,B). From there 1 µM and 5 µM of Dox were deployed to further study. It was shown that LC3B-II accumulated over time, but blocking SERTAD1 did not affect LC3B-II expression upon 1 µM of Dox treatment, while cleaved PARP activation was insignificant as predicted, suggesting the presence of autophagy-mediated cell death (Figure 3E). In contrast, upon 5 µM of Dox treatment, ablation of SERTAD1 significantly increased cleaved PARP but not LC3B-II expression over the 48-h time course, indicating the presence of apoptosis-mediated cell death (Figure 3F). It has been revealed that Dox elicits a cancer-cell-killing effect by regulating p53 activity [23], and the localization of p53 can trigger different types of PCDs [24]. For example, when p53 primarily localizes in nuclear, this action triggers autophagy, while apoptosis is induced when p53 is moved to cytoplasm. Indeed, our IF staining suggested a similar pattern (Supplementary Figure S1C,D) in which the co-localization of p53 and DAPI (nuclei marker) was increased in response to Dox 1 μM but decreased at 5 μM. Altogether, these results imply that different doses of Dox cause different types of PCDs and the suppressive role of SERTAD1 to apoptosis/anoikis in 2D and 3D cultures.

We were curious about how SERTAD1 contributes to Dox-induced apoptosis/anoikis in MCF7 cells. The two stable cell lines were treated with the two selected concentrations of Dox in the presence or absence of 25 μg/mL of chloroquine (CQ), a potent autophagy inhibitor. Consistent with the above results, treatment with 1 μM of Dox elevated the LC3B-II expression level, while the combination of 1 μM Dox and CQ induced PARP-dependent apoptosis/anoikis (Figure 3G). Adhering to previous findings [11], expression of SERTAD1 was markedly reduced in CQ-treated suspension cells (Figure 3G,H), which implies the repressive role of SERTAD1 in autophagy.

The combination of 5 μM Dox and CQ treatment greatly enhanced the cleavage of PARP compared with single treatments in the 3D condition. However, the combined treatment did not promote the apoptotic marker expression compared with single 5 μM Dox in SERTAD1-knockdowned cells (Figure 3H). In the single presence of CQ, cleaved PARP expression was significantly increased in SERTAD1-deficient cells compared to the controls in the 3D culture, indicating co-inhibition of autophagy, and SERTAD1 further forced the cells to enter anoikis (Figure 3G,H). Altogether, these data suggest the suppressive role of SERTAD1 on anoikis partly by inhibiting autophagy.

### 3.3. SERTAD1 Inhibits Dox-Induced Apoptosis/Anoikis by Regulating Mitochondrial and Lysosomal Activity

**Figure 4:**
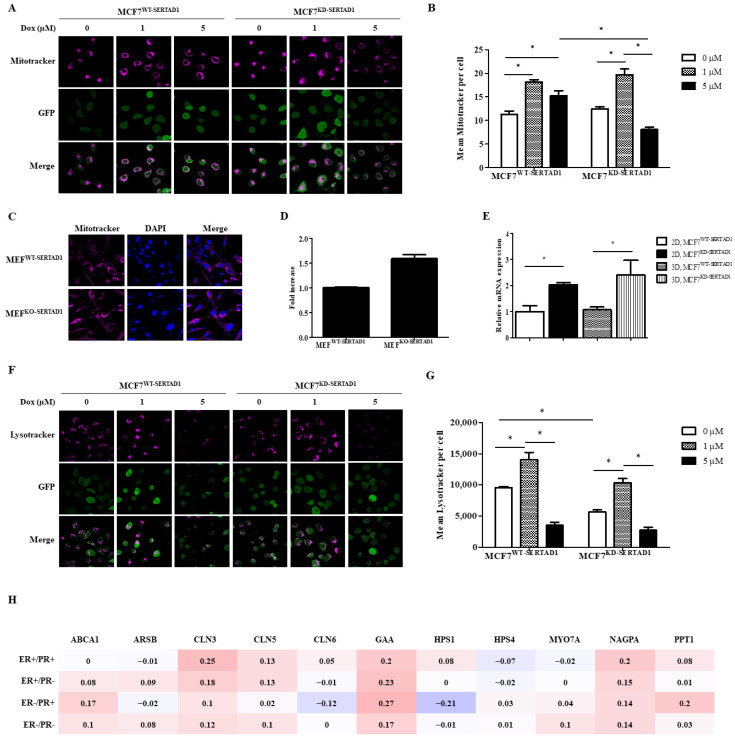
Inhibitory effect of SERTAD1 on apoptosis/anoikis partly via autophagy. (**A,B**) Mitochondrial contents of MCF7^WT-SERTAD1^ and MCF7^KD-SERTAD1^ cells in the presence of different concentrations of Dox. (**C,D**) Mitochondrial content of MEFs from SERTAD1^+/+^ and SERTAD1^−/−^ mice using Mitotracker. Mean Mitotracker per cell was analyzed using ImageJ program. (**E**) Levels of mtDNA in MCF7^WT-SERTAD1^ and MCF7^KD-SERTAD1^ cells. Images and data are representative of at least three independent experiments. * *p* < 0.05. (**F,G**) Lysosomal contents of MCF7^WT- SERTAD1^ and MCF7^KD-SERTAD1^ cells in the presence of different indicated concentrations of Dox in adherent cultures. Mean LysoTracker per cell was analyzed using ImageJ program. (**H**) Heat map of DNA microarray data in breast cancer patients by bc-GenExMiner v4.5 program showing the correlation between SERTAD1 and LYSOSOME_ORGANIZATION_AND_BIOGENESIS gene set.

Mitochondria and lysosomes are widely acknowledged as central regulators of PCDs [25,26,27,28]. To elucidate the mechanism by which SERTAD1-mediated Dox-induced cell death was triggered, we examined the potential role of SERTAD1 on mitochondrial and lysosomal activity. We found that in MCF7^WT-SERTAD1^ cells, mitochondria were well-preserved while that of MCF7^KD-SERTAD1^ cells were aggregated, accompanied by the increased intensity of mitochondrial staining (Figure 4A,B). Upon 1 μM of Dox treatment, mitochondria were redistributed to the cell periphery and increased in intensity. The abnormal increase of signal intensity could be due to the enhanced clearance of abnormal mitochondria through the high activation of mitophagy. Administration of 5 μM of the anti-cancer drug, in contrast, appeared to reduce the intensity of mitochondrial staining, suggesting different concentrations of Dox might kill cancer cells by activating different signaling cascades. Moreover, 5 μM Dox-treated SERTAD1-deficient cells were found to show significantly less mitochondrial activity than in control cells (Figure 4A,B), suggesting the potential involvement of activated mitoptosis. Suppressing SERTAD1 slightly increased mitochondrial activity in cancer cells (Figure 4A,B), but the phenomenon was clearly observed in mouse embryonic fibroblasts (MEFs) from KO-SERTAD1 mice compared with wild-type controls (Figure 4C,D, Supplementary Figure S2A). Previous findings suggested higher ROS levels in SERTAD1-inhibited breast cancer cells [10], which aligns with the higher mitochondrial content in SERTAD1-null MEFs and mtDNA in SERTAD1-blocked cells found in our study (Figure 4E).

Next, we stained MCF7^WT-SERTAD1^ and MCF7^KD-SERTADl^ cells with LysoTracker^®^ Deep Red to quantitate the number and spatial distribution of lysosomes. Indeed, the intensity was significantly enhanced in both cell lines treated with 1 μM of Dox, but diminished upon 5 μM of Dox treatment (Figure 4F,G), suggesting the induction of lysosomal membrane disruption. Moreover, the LysoTracker fluorescence signals were much stronger in MCF7^WT-SERTADl^ than MCF7^KD-SERTADl^ cells, suggesting lysosomal content was partly regulated by SERTAD1. The heat map generated from the DNA microarray data in breast cancer patients by bc-GenExMiner v4.5 program showed SERTAD1 positively co-expresses with the LYSOSOME_ORGANIZATION_AND_BIOGENESIS gene set (Figure 4H) and LYSOSOME gene set (Supplementary Figure S2B,C), suggesting the promoting role of SERTAD1 on lysosomal protein biosynthesis.

### 3.4. Dox-Triggered Cell Deaths Are LMP-Dependent and Ablation of SERTAD1 Reduces Protein Expression of Lysosomal Hydrolases

**Figure 5:**
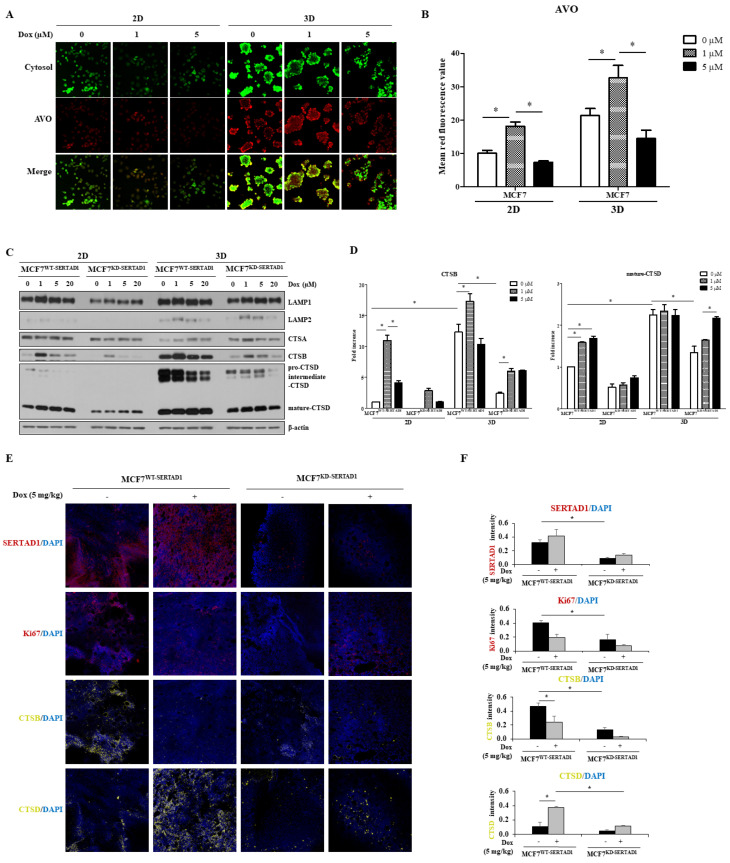
Effects of SERTAD1 on LMP induction and regulation of lysosomal hydrolases. (**A**,**B**) effect of Dox on lysosome integrity in MCF7 cells using AO staining. Cells were cultured in 2D and 3D conditions and then treated with indicated concentrations of Dox before being subjected to AO staining. AVOs per cell were analyzed using ImageJ. Images were randomly taken per plate. Data are representative of three independent experiments (n = 3). * *p* < 0.05. (**C**,**D**) Promoting role of SERTAD1 on lysosomal proteins. Total cellular extracts were prepared and expression levels of several lysosomal proteins, including LAMP1, LAMP2, CTSA, CTSB, and CTSD, were examined by immunoblotting. β-actin was used as the loading control. Blotting images and data are representative of at least three independent experiments with similar patterns. (**E**,**F**) Effect of SERTAD1 and Dox treatment on tumor growth and expression of CTSB and CTSD using IF staining. Images were randomly taken. Data are representative of at least three tumor samples. * *p* < 0.05.

LMP is described as a mechanism to induce cell death which causes the release of cathepsins (CTSs) and other hydrolases into the cytoplasm [27]. To evaluate the effect of Dox on LMP, AO staining was used to track the integrity of lysosomes. In response to 1 μM of Dox significant higher red punctate were observed, while the fluorescence intensity was strongly reduced upon 5 μM Dox treatment (Figure 5A,B), suggesting the induction of LMP by high concentrations of the anti-cancer drug under 2D and 3D conditions.

We next examined the expression levels of several prominent lysosomal proteins belonging to LAMP and CTS family. As shown in Figure 5C,D, with the exception of CTSA most of the tested proteins were more highly induced in the 3D culture than in the 2D condition. Moreover, inhibition of SERTAD1 suppressed the protein expression of all examined targets but not CTSA (Figure 5C,D). It could be seen that 1 μM of Dox upregulated the LC3B-II protein expression and enhanced the LysoTracker intensity in breast cancer cells, and the CTSB expression level was also increased at 1 μM of Dox while decreased at higher concentrations.

The findings were reconfirmed by IF staining in implanted tumors. The well-known marker for cell proliferation, Ki67, was chosen to evaluate the tumor proliferative activity. Indeed, Ki67 positive cells were much more abundant in wild-type control tumors compared to that of MCF7^KD-SERTAD1^ (Figure 5E,F), suggesting the promoting role of SERTAD1 in breast cancer cell proliferation. CTSB intensity levels were also significantly reduced in response to chemotherapy and suppression of SERTAD1 (Figure 5E,F), while that of mature CTSD were increased, which is consistent with our in vitro findings. It is noteworthy to mention that the detected CTSD fluorescent signals are likely the mature form of CTSD [29] unlike the pro and intermediate forms that are substantially decreased by SERTAD1 inhibition in 3D culture.

## 4. Discussion

Acquired chemoresistance of tumor cells is an unwanted consequence of cancer treatment. Dox elicits its therapeutic effects by generating ROS, which causes lipid peroxidation, compromising cell membrane and DNA and then inducing apoptosis [19]. Like other anti-cancer drugs, the effectiveness of Dox is limited by drug resistance, which can be categorized by acquiring incidents (i.e., present before treatment—intrinsic, or after treatment—acquired) [3]. High molecular and genetic heterogeneity of tumor cells which contain drug-resistant clones enables them to obtain drug resistance by positive selection depending on mutation rate and tumor size [30]. Alternatives of regulated and unregulated cell death rather than apoptosis may offer new strategies to overcome the intrinsic resistance. Besides, the elevated expression of oncogenic proteins and apoptosis inhibitors promotes anoikis resistance in cancer cells. In accordance with previous studies [10,11], our data also suggest the suppressive role of SERTAD1 on apoptosis/anoikis in response to Dox treatment, possibly via stabilizing XIAP [12,31].

Overcoming chemoresistance is particularly important for efficiently improving cancer therapies. MCF7 cells are highly resistant to various concentrations of Dox; however, the combination of inhibiting autophagy and the non-apoptosis-caused concentration of Dox could induce apoptosis and significantly reduce cell viability. The fundamental question of how autophagy dictates cell fate has been intensively debated. Thus, understanding connections and interactions between autophagy and apoptosis may allow for therapeutically manipulating autophagy in diverse disease contexts. Our results presented an intriguing phenomenon that different concentrations of Dox induced different types of cell deaths, prompting a potential clinical application with not only Dox but also other chemotherapeutic agents. High levels of autophagy (indicated by the elevated autophagic regulator, LC3B-II) found in 1 μM Dox-treated cells might implicate that the cells attempt to persist by inducing autophagy, and cell death occurs only when this effort desperately fails. When higher doses were administered, however, cells could not persist against such strong stresses and then entered apoptosis as the level of cleaved PARP was increased. Autophagy has cytoprotective effects under nutrient deprivation and other stresses; thus, autophagy-blocked cells undergo apoptosis. Our results also showed that co-inhibition of SERTAD1 and autophagy magnified apoptosis, indicating the concomitant targeting of SERTAD1 and other PCDs may help to improve cancer-killing strategies and overcome anoikis resistance.

Autophagy consists of several sequential steps, including sequestration, cargo transport to lysosomes, degradation, and ultimately the utilization of degraded products. CQ only targets autophagosome–lysosome fusion to inhibit autophagic flux [32]. When CQ was applied to cells cultured in 2D or 3D, a contrasting protein expression of SERTAD1 was found in these two culture conditions with results from the 3D culture well-aligned with our hypothesis and published findings. Validating cellular-signaling pathways using only the 2D cell culture may have a huge number of limitations due to the inability to represent or accurately mimic in vivo conditions [33]. This suggests that both types of cell cultures should be used to reliably confirm primary findings, especially with oncogenic SERTAD1, and with others. Besides, the presence and function of SERTAD1 in lysosome and autophagy, respectively, remains to be explored. SERTAD1 has been implicated in the metastatic spread of breast cancer cells by upregulation of the PI3K/AKT signaling cascade, partly via NEDD4-1-mediated PTEN ubiquitination [34]. Specifically, SERTAD1 suppresses the EMT process by affecting multiple EMT markers, such as E-cadherin, N-cadherin, and vimentin. Recently, circulating tumor cells (CTCs) have been considered as major driving forces of breast cancer metastasis [35]. CTC clusters undergo hypoxia while single CTCs are mostly normoxic [36]. Because SERTAD1 expression is increased in hypoxic conditions [37], it would be interesting to examine the role of SERTAD1 in the formation and shedding process of CTCs from the primary tumor.

As the blockade of SERTAD1 significantly decreased lysosomal contents, LMP might be strongly induced in the SERTAD1-knockdown cells. Additionally, only 1 µM Dox treatment increased the lysosomal contents of the cells, implying no initiation of LMP, which could be highly activated in the presence of 5 µM Dox. Autophagy and apoptosis can be activated by common upstream signals, implying the induction of the safeguarding processes depends on the cellular settings and context. On some occasions, cells can switch from autophagy to apoptosis and vice versa. In response to apoptosis-induced stimuli, cells can be killed even when apoptosis is inhibited, but the underlying mechanisms are not clear. CTSB has been proposed to be a molecular link between autophagy and apoptosis in which suppression of CTSB inhibits apoptosis but not autophagy [38]. Therefore, we suspect CTSB may dictate Dox-induced autophagy- and apoptosis-mediated cell death in breast cancer cells. Another possibility is that 1 µM-treated cells may compromise proapoptotic caspases due to the inhibition of BAX and BAK, and calpain-like protease [39,40]. The non-apoptotic death found in our study may be BECLIN1- and ATG5-dependent. Since p53 is one of major upstream molecules triggering LMP [41], the low and high dose of Dox used in this study might distinctly induce the localization of p53, leading to different mechanisms that decide cell fate. Although SERTAD1-mediated activity of lysosomal proteins was not investigated, SERTAD1 may also regulate the activity of affected lysosomal hydrolases, but this needs further study.

Mitochondria and lysosomes, similar to other cellular organelles, are essential to numerous biological processes in living organisms. The aberrant expression of either mitochondrial proteins or lysosomal counterparts negatively affects cellular integrity and function; thus, the biogenesis of these two organelles is tightly controlled by cells. Results from this study revealed a novel physiological function of SERTAD1 in homeostatic maintenance, but the underlying mechanism remains a gray area. Cathepsins play important roles in cellular homeostasis, cell proliferation, tumor progression, drug resistance, metastasis, and others [42,43]. There are several major transcriptional regulators in lysosomal biogenesis, such as *TFEB/TFE3*, *STAT3*, *MYC,* and *ZKSCAN3*, and other epigenetic regulators [8]. Since inhibition of SERTAD1 only reduces lysosomal hydrolases, and it was proposed that activated STAT3 is responsible for regulation of lysosomal proteases by binding to promoters of downstream target genes [8,44], we propose that indirect activation of STAT3 by SERTAD1 may account for changes in lysosomal protein expression.

**Figure 6:**
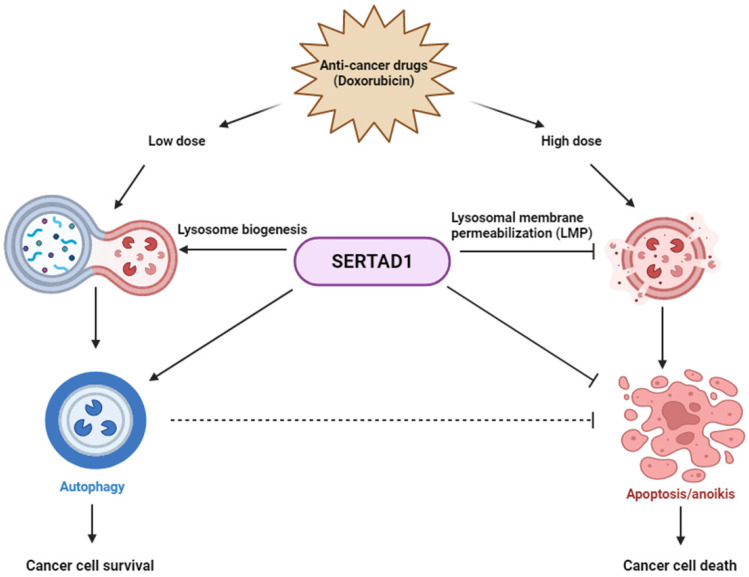
Summary model. SERTAD1 sensitizes cancer cells to anti-cancer drugs and affects lysosome biogenesis and LMP.

In conclusion, our study is supported by multiple lines of evidence visualized in Figure 6. We determined that distinct PCDs can be induced by different doses of Dox; SERTAD1 suppresses sensitivity of MCF7 to anti-cancer drugs and regulates the synthesis of lysosomal proteins.

## Data Availability

Not applicable.

## Supplementary Materials

The following supporting information can be downloaded at: https://www.mdpi.com/article/10.3390/biomedicines10051148/s1, Figure S1: Effect of SERTAD1 on the expression of LC3B and p53 in the presence of Dox. A-B, immunofluorescence images of LC3B in response to indicated concentrations of Dox in SERTAD1-deficient and wild-type cell lines. C-D, immunofluorescence staining of p53 using confocal microscope. Signal intensity was analyzed using ImageJ. * *p* < 0.05; Figure S2: Effect of SERTAD1 on mitochondrial and lysosomal activity. A, genotyping of SERTAD1 wild-type (WT) and SERTAD1 knockout (KO) C57BL/6 mice. Four representative MEF samples from WT- and KO-SERTAD1 mice were collected and subjected to agarose gel electrophoresis. B-C, heat map of DNA microarray data in breast cancer patients by bc-GenExMiner v4.5 program to show the correlation between SERTAD1 and REACTOME_MITOCHONDRIAL_BIOGENESIS gene set (B) and LYSOSOME gene set (GO:0005764) (C).
