# Supplementary material for: SERTAD1 Sensitizes Breast Cancer Cells to Doxorubicin and Promotes Lysosomal Protein Biosynthesis
Source: Biomedicines. 2022 May 17;10(5):1148. doi: 10.3390/biomedicines10051148 (PMC9139069; doi:10.3390/biomedicines10051148)
Supplement: Supplementary file 1 [file biomedicines-10-01148-s001.zip › biomedicines-1699526-supplementary.pdf]

Supplementary-Figure S1

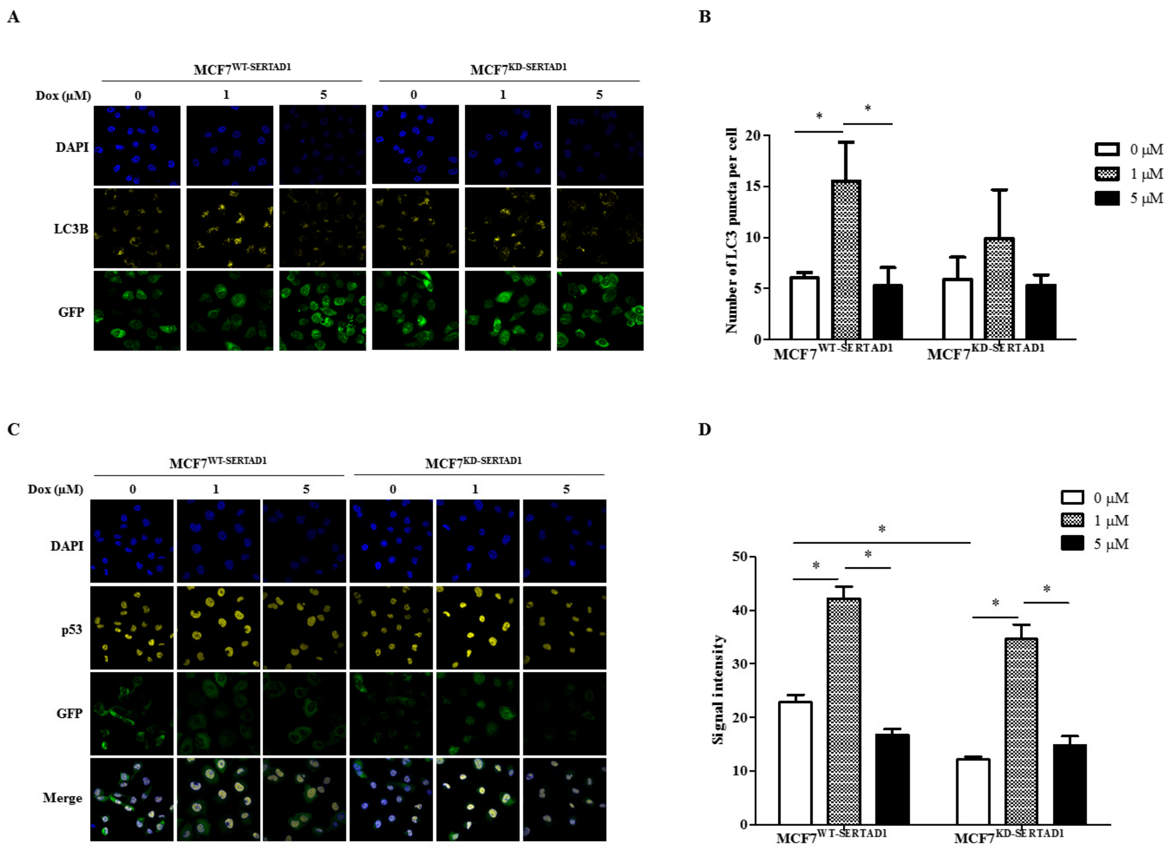

Figure S1. Effect of SERTAD1 on the expression of LC3B and p53 in the presence of Dox. **A-B**, immunofluorescence images of LC3B in response to indicated concentrations of Dox in SERTAD1-deficient and wild-type cell lines. **C-D**, immunofluorescence staining of p53 using confocal microscope. Signal intensity was analyzed using ImageJ. $*P < 0.05$.

Supplementary-Figure S2

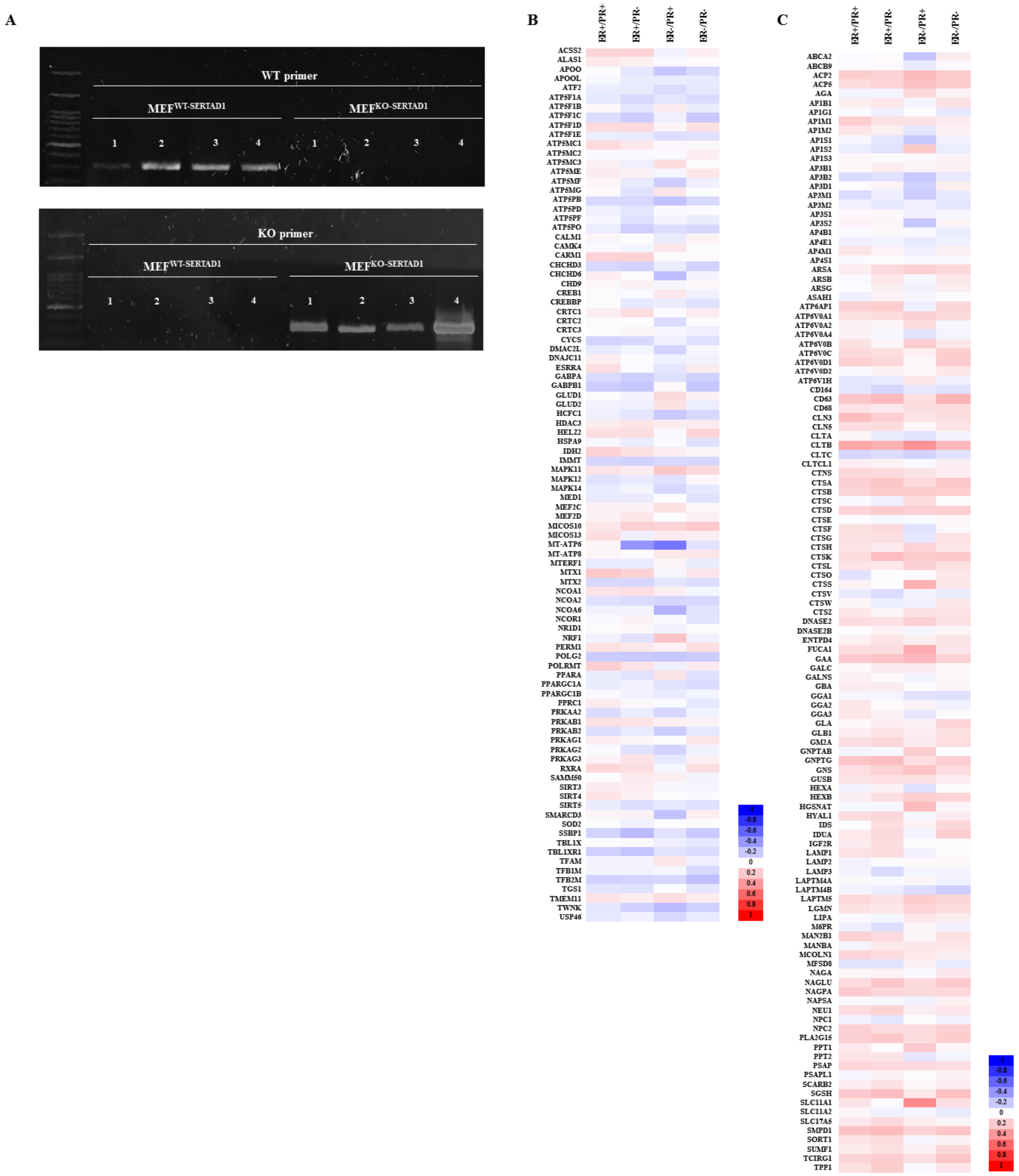

Figure S2. Effect of SERTAD1 on mitochondrial and lysosomal activity. **A,** genotyping of SERTAD1 wild-type (WT) and SERTAD1 knockout (KO) C57BL/6 mice. Four representative MEF samples from WT- and KO-SERTAD1 mice were collected and subjected to agarose gel electrophoresis. **B-C,** Heat map of DNA microarray data in breast cancer patients by bc-GenExMiner v4.5 program to show the correlation between SERTAD1

and REACTOME_MITOCHONDRIAL_BIOGENESIS gene set **(B)** and LYSOSOME gene set (GO:0005764) **(C)**.
